# Molecular Characterization of Circulating Tumor Cells in Human Metastatic Colorectal Cancer

**DOI:** 10.1371/journal.pone.0040476

**Published:** 2012-07-10

**Authors:** Jorge Barbazán, Lorena Alonso-Alconada, Laura Muinelo-Romay, María Vieito, Alicia Abalo, Marta Alonso-Nocelo, Sonia Candamio, Elena Gallardo, Beatriz Fernández, Ihab Abdulkader, María de los Ángeles Casares, Antonio Gómez-Tato, Rafael López-López, Miguel Abal

**Affiliations:** 1 Translational Laboratory, Medical Oncology Department, Complexo Hospitalario Universitario de Santiago de Compostela/SERGAS, Santiago de Compostela, Spain; 2 Department of Pathology, Complexo Hospitalario Universitario de Santiago de Compostela/SERGAS, Santiago de Compostela, Spain; 3 School of Mathematics, Universidad de Santiago (Campus Vida), Santiago de Compostela, Spain; National Cancer Institute, United States of America

## Abstract

Metastatic colorectal cancer (mCRC) relies on the detachment of aggressive malignant cells from the primary tumor into the bloodstream and, concordantly, the presence of these Circulating Tumor Cells (CTC) is associated with a poor prognosis. In this work, the molecular characterization of CTC from mCRC patients was approached, with the aim of understanding their biology and improving their clinical utility in the management of colorectal cancer patients. For this, EpCAM-based immunoisolation of CTC was combined with whole transcriptome amplification and hybridization onto cDNA microarrays. Gene expression data from mCRC patients, once the background of unspecific immunoisolation from a group of controls had been subtracted, resulted in 410 genes that characterized the CTC population. Bioinformatics were used for the biological interpretation of the data, revealing that CTC are characterized by genes related to cell movement and adhesion, cell death and proliferation, and cell signalling and interaction. RTqPCR on an independent series of mCRC patients and controls was used for the validation of a number of genes related to the main cellular functions characterizing the CTC population. Comparison between primary carcinomas and lung and liver metastases further involved the CTC-genes in the promotion of metastasis. Moreover, the correlation of CTC-gene expression with clinical parameters demonstrated detection and prognosis significance. In conclusion, the molecular characterization of CTC from mCRC patients and the identification of diagnostic and prognostic biomarkers represent an innovative and promising approach in the clinical management of this type of patients.

## Introduction

Colorectal cancer (CRC) is the third most commonly diagnosed cancer in males and the second in females, with over 1.2 million new cancer cases estimated to have occurred worldwide in 2008 [1]. Clinically, distant tumor dissemination and metastasis are the most important factors in prognosis: whereas non-invasive stage I carcinomas present a 90% five year-survival, stage IV carcinomas with distant metastasis correlate with a dramatic drop to a 10% survival rate [2]. Consequently, new therapeutic strategies improving the efficacy against metastatic disease and accurate biomarkers for the follow-up of CRC patients are major challenges, together with early detection and screening in high-risk populations.

The spread of cancer relies on the detachment of aggressive malignant cells from the primary tumor into the bloodstream as a principal source of the further metastasis [3]. It is widely accepted that Circulating Tumor Cells (CTC) own or acquire the ability to evade the host immune system and to reach a distant organ, usually the liver in CRC, where they establish a secondary tumor growth site in a highly inefficient but dramatic process [4]. Concordantly, the presence of CTC in peripheral blood has been associated with poor prognosis in different types of cancer, including CRC [5], [6]. As presumptive founders in the generation of metastasis, CTC are becoming a field of interest, and the understanding of their biology may open new perspectives in oncology. Concerning their molecular characterization, in the last few years a number of groups have presented expression data focused on specific genes or signalling pathways related to cancer for the improvement of sensitivity and specificity of the detection [7], [8]. Smirnov et al. approached the profiling of CTC through a miscellaneous breast, prostate and colorectal metastatic perspective [9]. In addition, data is emerging on the possibility of studying the biology and the utility to evaluate targeted therapies based on the genomic profiling of CTC [10], [11].

Within this scenario, defined by a limited efficacy of current chemotherapies in the treatment of metastatic CRC (mCRC) and CTC as key players in the management of metastatic disease, specific molecular profiling of the CTC population was approached. The combination of CTC EpCAM-based immunoisolation and accurate extraction of RNA from the very small number of CTC plus whole transcriptome amplification, made it possible to hybridise cDNA from the CTC population onto gene-expression microarrays. By applying this procedure to a group of mCRC patients compared to the background of unspecific isolated hematopoietic cells, the population of immunoisolated CTC was profiled specifically. In addition to the molecular characterization of CTC for the understanding of the biology of a main source of metastasis in CRC, these data provided potential therapeutic targets and diagnostic/prognostic biomarkers.

## Results

### CTC Immunoisolation and Molecular Profiling

**Figure 1:**
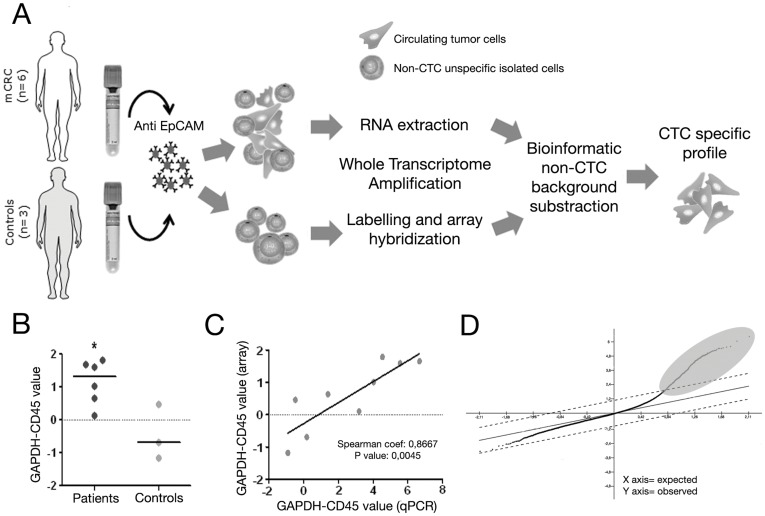
CTC gene expression profiling methodology. (**A**) Schematic representation of the procedure used for CTC molecular characterization. CTC were isolated from 7.5 mL of peripheral blood by immunomagnetic separation using anti-EpCAM coated magnetic beads. Isolated cells were subjected to a RNA extraction followed by a whole transcriptome amplification process (WTA). Finally amplified cDNA was hybridized onto Agilent gene expression arrays. (**B**) GAPDH-CD45 levels in controls and mCRC patients measured by real time PCR. Horizontal bars represent the median value of each group (*p<0.05). (**C**) Spearman correlation analysis between GAPDH-CD45 levels obtained both from post-array data and qPCR previous to WTA amplification. (**D**) SAM analysis output graph showing gene expression differences between the group of patients and controls. Dots highlighted correspond to genes with statistically significant increased levels of expression in the group of patients compared to the control background, being considered to characterize the CTC population from mCRC.

The procedures for CTC immunoisolation, RNA extraction and amplification for hybridisa;tion onto cDNA microarrays are depicted in Figure 1A. Briefly, CTC were immunoisolated from 7.5 ml of peripheral blood from stage IV mCRC patients (n = 6; Table S1). Magnetic beads were used, which were coated with a monoclonal antibody towards the human Epithelial Cell Adhesion Molecule (EpCAM), a surface molecule highly expressed in epithelium-originated tumors such as CRC. RNA from isolated CTC was purified using a kit specifically designed for low abundance samples. In parallel, the same protocol was applied to blood samples from healthy donors (n = 3) to establish the baseline of background from unspecific non-CTC immunoisolation. Prior to gene expression analysis, the presence of isolated CTC was confirmed by direct immunofluorescence visualization using a cocktail of antibodies against cytokeratins 8, 18 and 19 (Figure S1), and by a combination of two biomarkers validated for the accurate quantification of CTC in mCRC patients (GAPDH-CD45; [12]) (Mann-Whitney U, p-value <0.05) (Figure 1B). Moreover, the accuracy of the methodology assessed as the recovery rate during immunoisolation resulted in a median of 91.56% (Methods S1).

In order to characterize the CTC population isolated from mCRC patients, the methodology described by Gonzalez-Roca et al. was adapted, for accurate gene expression profiling of very small cell populations [13]. Basically, purified RNA was further treated with DNaseI, amplified using the WTA2 whole transcriptome amplification method, and complementary DNA was labeled and hybridized onto Agilent expression arrays (Figure 1A) (Gene Expression Omnibus, GEO. Accession number: GSE31023). After the initial pre-processing of raw data, an average of 21,070 spots were filtered according to the criteria described in Materials & Methods, which represented 47.35% of the spots in the microarray with a maximum of 32,443 and a minimum of 13,247. Upon filtration, the % coefficient of variation (CV) for replicated probes ranged from 5.98% to 12.13%. Normalization within each microarray was carried out using the Loess method, which assumes that most genes in microarrays are not differentially expressed in comparison to the control, so normalization among all microarray data was performed by the Aquantile method implemented in the Limma package of the R statistical software. This method ensured that the A values (average intensities) had the same empirical distribution across microarrays whilst leaving M values (log-ratios) unchanged. Correlation analysis of RTqPCR GAPDH-CD45 levels before and after amplification indicated a robust linearity during the process of transcriptome amplification (Spearman coefficient: 0.8667; p-value <0.005) (Figure 1C).

**Figure 2:**
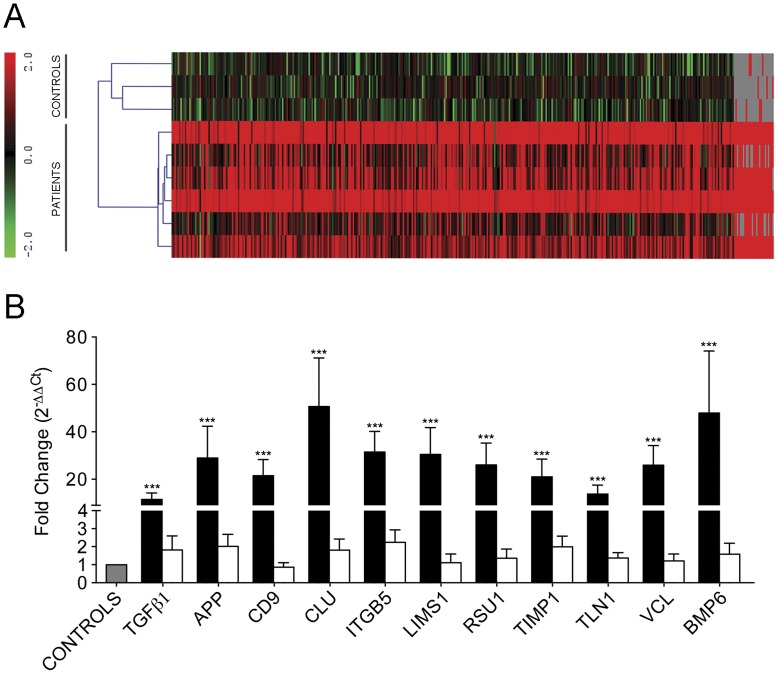
Gene expression analysis and validation. (**A**) Hierarchical clustering of differentially expressed genes between patients (n = 6) and controls (n = 3) (CTC specific genes). (B) RTqPCR validation of eleven genes selected from array data. CD45-normalized fold change differences between mCRC patients (n = 20) and healthy controls (n = 10) (grey bar) in the CTC enriched fraction (black bars; ***p<0.0001). Of note, no differences were observed between mCRC patients (n = 5) and controls (n = 5) in the remaining fraction after CTC immunoisolation (white bars).

The next step involved gene-expression profiling of CTC isolated from mCRC patients against the background of contaminating cells during the immunoisolation. For this, normalized gene-expression intensities from mCRC patients and from the group of controls were processed using MeV (MultiExperiment Viewer) software (see Methods S1). The signals obtained from healthy controls were considered as the background from non-specifically isolated blood cells, which were mainly lymphocytes. The signal obtained from mCRC patients represented the sum of this non-specific background plus the specific gene-expression pattern of the CTC. By subtracting this background, the contaminating non-CTC population was removed, and the resulting genes showing statistically significant expression were considered to characterize the CTC population from mCRC patients (Figure 1A). Concordantly, all significant genes presented positive expression in mCRC patients upon subtraction of the background from healthy donors (Figure 1D), which was consistent with the presence of CTC only in the mCRC samples. This strategy led to the identification of a final set of 410 genes that were specific to the CTC population (Table S2), with hierarchical clustering analysis clearly discriminating between mCRC patients and controls (Figure 2A).

Bioinformatic analysis with Ingenuity Pathway Analysis (IPA) and Genecodis (for Gene Ontology) software served to interpret the resulting list of genes characterizing the CTC population. The main cellular functions defined by these CTC-specific genes were related to cell movement, cell adhesion, cell death and proliferation, cell-cell signalling and interaction, and cytoskeleton reorganization (Figure S2A and C). Interestingly, the IPA analysis of canonical pathways, which was representative of these genes, also highlighted a number of known signaling pathways involved in cell migration/invasion and cell adhesion, as Protein Kinase A, RhoA, Integrins, ILK, or Actin cytoskeleton signaling molecules (Figure S2B). Moreover, the analysis of gene-gene interactions rendered biological networks that characterize the CTC population from mCRC patients. Among them, cancer and cellular movement and morphology were found to be the principal events associated with a CTC phenotype, while this interpretation might be biased by the important contribution of cancer to databases (Figure S3). All of these analyses point to a balance among the genes underlying cell survival, interaction with the environment and cellular movement as the fundamental biological processes that must converge in the population of CTC for the successful development of metastasis in CRC.

### Real-Time Quantitative PCR Validation and Identification of Diagnostic and Prognostic Biomarkers

The next step involved the RTqPCR validation of eleven genes presenting high log2 ratios in mCRC patients and a functional relevance in the biological processes characterizing the CTC population in an independent series of mCRC patients and controls. The specific expression of these genes was evaluated by comparing the CTC populations from the group of mCRC patients (n = 20) with the background from healthy controls (n = 10), once they had been normalized to CD45 as a marker of unspecificity during CTC isolation. These genes included APP, CLU and TIMP1, which are associated with cell death and anti-apoptotic activity; VCL, ITGB5, BMP6 and TGFβ1, which are genes involved in cell migration and invasion and cellular morphology; and TLN1, ITGB5, LIMS1, RSU1 and CD9, which are associated with cell adhesion. As observed in Figure 2B (black bars), all of the selected candidate genes were validated in this new series of samples by RTqPCR with significant differences between the group of patients and the group of controls (p-value <0.0001).

Importantly, to ensure that the expression of the selected genes was characteristic of CTC and not an artefact due to the variation of gene expression in lymphocytes in cancer patients, their expression was compared in the remaining non-isolated fraction upon CTC enrichment in a set of mCRC patients (n = 5) and controls (n = 5). As shown in Figure 2B (white bars), no differences were found between both groups for the eleven candidate genes, reinforcing their specificity of expression in the CTC population. When five samples in the CTC isolated fraction were randomly selected, significantly higher levels were consistently found in mCRC samples, demonstrating that the lack of differences between patients and controls in the non-isolated fraction was not due to sample size artefacts (data not shown).

**Figure 3:**
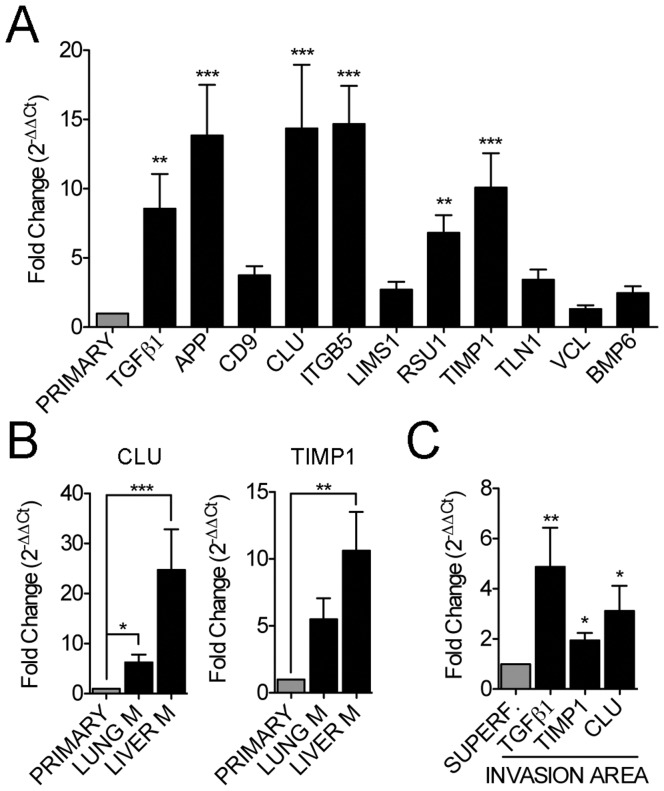
Gene expression of selected genes in primary tumors and metastases. (**A**) Gene expression differences between lung and liver metastases from CRC patients (n = 14) and primary tumors (n = 14) for the eleven validated genes. (**B**) Differences in gene expression for CLU and TIMP1 between lung (n = 7) and liver (n = 7) metastases (M) compared with the primary tumor. (**C**) Specific up-regulation of TGFβ1, TIMP1 and CLU in the invasive front of CRC primary tumors (n = 14) compared to the non-invasive area (*p<0.05; **p<0.01; ***p<0.001).

To further evaluate the involvement of the candidate genes in the metastatic potential of CTC in CRC, their expression was compared in a series of primary carcinomas (n = 14) and lung (n = 7) and liver (n = 7) metastases. As shown in Figure 3A, all of the selected genes were up-regulated in metastases compared to primary lesions, with seven out of the eleven genes presenting statistical significance. Among them, CLU and TIMP1 presented a specific up-regulation in liver metastasis, suggesting a potential role for these genes in the tissue-specific ability of mCRC CTC to colonize the liver (Figure 3B). In addition, three out of the eleven selected genes demonstrated a significantly increased expression at the invasive front of the primary tumors compared to the paired non-invasive superficial area, suggesting a link to the acquisition of an aggressive phenotype (Figure 3C). All of these results validated the strategy to molecularly profile the CTC in mCRC patients, confirming the presence of the CTC population and the efficient immunoisolation of CTC from mCRC patients, as well as the capability to specifically characterize these cells at the molecular level.

Finally, the molecular profiling of CTC should result in the identification of potentially reliable biomarkers for the detection of the CTC population from mCRC patients. To evaluate this point, the accuracy of the RTqPCR validated genes in the detection of metastatic disease was evaluated in the series of mCRC patients and controls. As shown in Table 1, all of the validated genes presented excellent discriminating values in terms of AUROC (values ranging from 0.94 to 1; p-value <0.0001). Moreover, and with the exception of CD9 and TLN1, the selected biomarkers demonstrated an excellent behavior as predictors of disease progression. These data guarantee further studies in large cohorts of patients for the use of these biomarkers in the clinical setting.

**Table 1:** Diagnostic and prognostic value of array validated genes.

	ROC curve analysis			Survival analysis		
Gene name	AUC	p-value	95% CI	Cutoff value	p-value	Median survival time (months)^1^
**TGFβ1**	1.00	<0.0001	1.00 to 1.00	5.5	0.045	10.00 *vs* 5.93
**APP**	1.00	<0.0001	1.00 to 1.00	3.5	0.004	10.33 *vs* 5.93
**CD9**	0.98	<0.0001	0.93 to 1.02	2	0.067	10.00 *vs* 8.06
**CLU**	0.95	<0.0001	0.87 to 1.03	5.5	0.001	11.43 *vs* 5.60
**ITGB5**	0.95	<0.0001	0.86 to 1.04	1.2	0.005	10.33 *vs* 4.4
**LIMS1**	0.98	<0.0001	0.93 to 1.03	2.5	0.007	11.43 *vs* 5.60
**RSU1**	0.94	0.0001	0.86 to 1.02	5	0.003	11.43 *vs* 5.60
**TIMP1**	0.97	<0.0001	0.91 to 1.03	3	<0.0001	10.33 *vs* 5.60
**TLN1**	1.00	<0.0001	1.00 to 1.00	4	0.218	10.00 *vs* 5.93
**VCL**	1.00	<0.0001	1.00 to 1.00	4	0.001	10.33 *vs* 5.60
**BMP6**	1.00	<0.0001	1.00 to 1.00	−1	0.005	12.36 *vs* 6.43

ROC curve analysis results are represented. Area Under ROC Curve (AUC), statistical p-value and 95% Confidence Intervals (CI) are shown. Kaplan-Meier Progression Free Survival (PFS) analysis is presented. Cutoffs refer to best-fit values. Survival p-values result from Log-Rank test.

1 Below marker cutoff group versus over marker cutoff group.

## Discussion

Molecular profiling is widely employed as a powerful strategy to characterize specific types of tumors, specific subtypes of carcinomas or specific events associated with carcinogenesis. In addition to contribute to the understanding of molecular events associated with the genesis and progression of cancer, gene-expression profiling has led to the identification of therapeutic targets and biomarkers in an effort to improve the management of cancer patients at the clinical setting. In this work, we aimed to tackle with a very specific and attractive population of tumor cells that are at the origin of metastasis, the circulating tumor cells (CTC). A combination of CTC immunoisolation, accurate RNA extraction from very low number of cells, whole-genome amplification and massive gene-expression profiling for the characterization and interpretation of the biology of CTC in metastatic colorectal cancer is presented here. To technically validate this strategy, the profiling of CTC from mCRC patients was approached by subtracting the background of non-specific isolation from a group of healthy controls. The next challenge is to compare the gene-expression profile of the CTC population with the primary CRC lesions and with the overt metastases, in order to better understand the mechanisms of adaption of tumor cells during the process of metastasis and the crosstalk with the environment. The subtraction of the background from a group of controls was also considered to be more relevant than the background from the same mCRC patients as it would require two rounds of CTC isolation within the same samples, representing a significant source of technical artefacts. Differences in the background could not be excluded due to the systemic metastatic disease, although the analysis of the remaining fraction after CTC isolation did not render any differences within the selected genes.

Regarding immunoisolation, and although CTC enrichment with EpCAM-coupled antibodies has demonstrated to be superior to other cytometric methods and a reliable method for CTC detection in mCRC patients [14], this CTC capture procedure has raised some debate due to the reliance of this technique on the expression of EpCAM. Initially described 30 years ago as a dominant antigen in human colon carcinoma tissue, it is assumed that a decreased expression of epithelial markers occurs during the epithelial to mesenchymal transition (EMT) associated with tumor invasion [15]. Importantly, EpCAM is apparently needed to maintain distinct cancer cell attributes and, potentially, the cancer stem cell phenotype [16]. CD133+ cells, currently one of the best markers for the characterization of colon cancer stem cells and an independent prognostic marker that correlates with low survival, are positive for EpCAM [17]. Likewise, antibodies against EpCAM can efficiently target colorectal Tumor-Initiating Cells [18], conferring a considerable value to the EpCAM-isolated CTC population in terms of therapeutic intervention. The data presented here conclusively demonstrated the effective isolation of CTC from mCRC patients.

**Figure 4:**
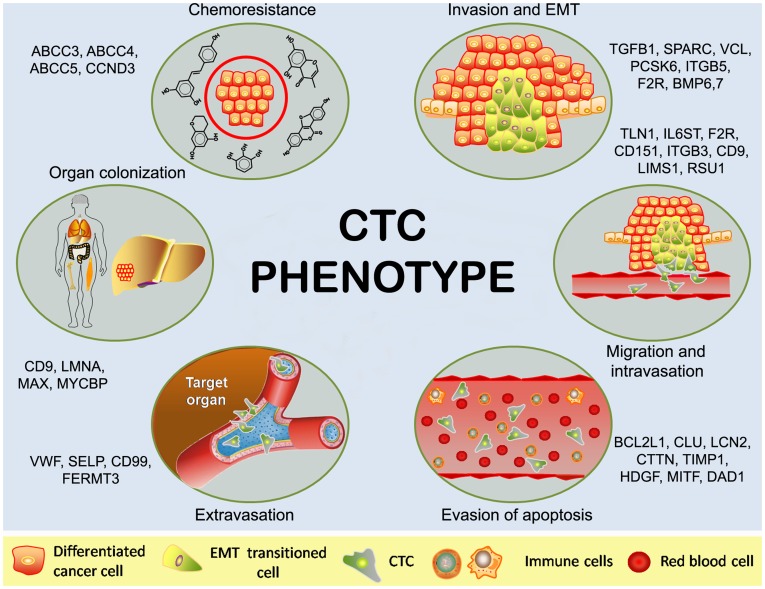
Schematic representation of the proposed CTC phenotype from mCRC patients. Of the resulting microarray dataset, some of the most relevant genes are showed in relation with certain features of the process of metastasis.

A main achievement of this work was the translation of the method described by Gonzalez-Roca et al. for the accurate expression profiling of very small cell populations [13], into a clinically relevant cell population. Whole transcriptome amplification allowed the characterization of the CTC population isolated from metastatic CRC patients at the molecular level. To date, the majority of studies have described the expression levels of a limited number of genes in different CTC populations, principally for detection purposes [19], [20]. The approach used here allowed the molecular profiling of CTC from mCRC, and their definition as a population of cells with presumed migratory and adhesive capabilities. This is consistent with a subpopulation of tumor cells that must acquire an aggressive and invasive phenotype allowing dissociation from the primary lesion, leading to the invasion of the surrounding stroma and their intravasation and survival in the blood flow. In addition, these cells may also extravasate and successfully implant in the distant tissue target to generate a micrometastasis [19]. The list of genes phenotypically characterizing the CTC population from mCRC patients includes candidates related to all the above described functions (Figure 4), warranting further studies to define their implication in the metastatic behavior of the CTC population in CRC. Genes such as VCL, ITGB5, BMP6 or TGFβ1 have been associated with the acquisition of an invasive phenotype [8], [21], [22], in part through EMT. Likewise, TLN1, APP, CD9, LIMS1 and RSU1 have been related to adhesion and migration, with CD9 modulating the localization of TLN1, a critical regulator of integrin activation, to focal adhesions [23], or LIMS1 being associated with cell adhesion and integrin signaling, and, in particular, with RSU1 and the Ras pathway during cell migration [24]. A key step in the process of cancer dissemination is the ability to migrate and to progress towards and intravasate nearby blood capillaries. Once in the circulation, CTC must overcome the host immune system by enhancing their apoptosis resistance among other mechanisms. Genes like TIMP1 and CLU are key molecules related to this process [25]. Interestingly, TIMP1 has been linked to the process of anoikis resistance in different cancer models, enabling the evasion of cell death in the absence of substrate anchorage [26]. Upon reaching a target organ, CTC must be able to extravasate and form colonies, and CD9 has been described as critical to the process of implantation and micrometastases formation associated with stem cell attributes, which is related to these events [27]. Interestingly, a significant overlap was found with a liver-specific metastasis signature in CRC [28] (see Table S4), suggesting an active implication in the tropism and micrometastasis potential of CTC to this organ in CRC. In particular, and consistent with the correlation of TIMP1 expression in CTC and liver metastasis, it has been described as a regulator of the liver microenvironment, increasing the susceptibility of this organ to tumor cells [29]. Overall, the involvement of the selected genes in the metastasis capacities of CTC has also been reinforced by their up-regulation in lung and liver metastases compared to primary colorectal carcinomas. *In vitro* primary CTC cultures and *in vivo* models for CTC in CRC will definitely provide researchers with robust evidence to validate the role of these genes in the ability of CTC to generate micrometastases, and to define strategies specifically targeting this population of metastatic CTC.

Likewise, in terms of biomarkers for the management of mCRC patients, the molecular profiling of CTC from mCRC patients rendered valuable tools for the detection and quantification of metastatic disease, as well as for the prediction of disease progression. The high specificity and sensitivity demonstrated by the above described validated genes in the CTC population from mCRC patients warrant the study of these biomarkers in the evaluation of therapeutic responses or in the selection of patients for targeted therapies.

In conclusion, this study described, to our knowledge, the first specific molecular profiling of CTC isolated from mCRC patients. This gene-expression analysis was applied to the characterization of this specific population with presumed adhesive, migratory and invasive capabilities, as well as a modulated response to cell death for the successful completion of the process of metastasis. Moreover, the identification of therapeutic strategies specifically targeting the CTC population should improve the efficacy in the eradication and prevention of CRC metastasis, while an armamentarium of highly specific and sensitive valuable markers should impact on the management and follow-up of metastatic CRC patients.

## Materials and Methods

### Patients

All participants signed an informed consent specifically approved for this study by the Ethical Committee of the Complexo Hospitalario Universitario of Santiago de Compostela (code of approval: 2009/289). Inclusion criteria for mCRC patients were the presence of measurable mCRC and an Eastern Cooperative Oncology Group (ECOG) performance status not greater than 2. Healthy controls with an absence of a previous cancer episode and an age matched with patients were selected. Detailed information about patients included in the analysis is available in Table S1.

### CTC Immunoisolation

CTC were isolated by using the CELLection™ Epithelial Enrich kit (Invitrogen, Dynal) according to manufacturers’ instructions. Briefly, 7,5 mL of blood from mCRC patients and healthy controls were incubated for 30 minutes at 4°C with 100 µl of magnetic beads. After washing, CTC coupled to the magnetic beads were directly resuspended in 100 µl of RNAlater® solution (Ambion) and stored at −80°C until processed for RNA extraction.

### Gene Expression Analysis

Total RNA from CTC was extracted with the QIAmp viral RNA mini kit (Qiagen), specifically designed for very low cellularity samples. Purified RNA was next subjected to a whole transcriptome amplification reaction (WTA2, Sigma Aldrich), Cy3 labeled and hybridized onto Agilent 4×44 k gene-expression arrays. Signal processing and filtering as well as gene expression data analysis are described in detail in Supplemental Material. Gene expression data is accessible at the NCBI Gene Expression Omnibus (GEO) database (Accession Number: GSE31023).

### Real-Time Quantitative PCR Validation

RTqPCR validation was performed in an independent set of 10 healthy controls and 20 stage IV metastatic colorectal cancer patients (see Table S1). CTC were isolated as described, and RNA was purified with a RNA carrier to improve yield and stability. cDNA was synthesized by using SuperScriptIII chemistry (Invitrogen) following manufacturer’s instructions. To further optimize the sensibility of detection, we performed a preamplification step by using the TaqMan® PreAmp Master Mix kit (Applied Biosystems) with 14 reaction cycles. Preamplified products were subjected to TaqMan® real-time PCR amplification for eleven candidate genes (see Table S3 for assay details).

In addition, we evaluated by RTqPCR the expression levels of the candidate genes in the cellular fraction remaining upon CTC immunoisolation, in 5 mCRC patients and 5 healthy controls. Briefly, we performed a red blood lysis in the fraction remaining after EpCAM immune-bead incubation and CTC isolation, and RNA was purified from the remaining nucleated blood cells with the RNeasy mini kit (Qiagen). RNA quantity and purity were assessed by NanoDrop measurement, cDNA was synthesized using MuLV reverse transcriptase system (Applied Biosystems) and TaqMan® qPCR was performed for the eleven candidate genes.

Expression values for each gene were normalized to CD45 as a marker of non-specific isolation. Fold change differences between patients and controls were statistically analyzed with the GraphPad Prism software, applying the Mann-Whitney non-parametric t-test and considering a p-value<0.05 as significant.

### Gene Expression Analysis in Primary Tumors and Metastases

Primary colorectal carcinomas (n = 14) and metastasis (liver metastasis, n = 7; lung metastases, n = 7) were processed by the Pathology Department of the Complexo Hospitalario Universitario of Santiago de Compostela. The superficial non-invasive zone and the deep invasive area of the primary tumors were macroscopically dissected, ensuring similar tumor percentages. RNA was purified (TRIZOL reagent, Invitrogen; RNeasy kit, Qiagen), cDNA was synthesized (MuLV reverse transcriptase, Applied Biosystems), and gene expression was evaluated (TaqMan RTqPCR, Applied Biosystems). Data was represented as fold change relative to the expression in the superficial non-invasive area. GAPDH was used as loading control. Non-parametric Mann-Whitney t-test was used for statistical significance considering p-value <0.05.

### Area under the ROC Curve (AUROC) and Kaplan-Meier Analysis

The accuracy of the eleven candidate genes as biomarkers for diagnosis was evaluated with ROC curves, while Kaplan-Meier curves were built for their evaluation as markers for prognosis. Cutoff values were set as the best-fit values for each marker. Patients Progression Free Survival times were established as the time elapsed between chemotherapy line start and disease progression evaluated by imaging, or patient dead by any cause.

## Supporting Information

Figure S1 **Representative images of epithelial cells isolated from mCRC patient’s blood**. Cells were stained with a cocktail of antibodies against citokeratins (left panels) and visualized in clear field (right panels).
(TIF)

Figure S2 **Gene interaction analysis.** Principal molecular and cellular functions (**A**) and signaling pathways (**B**) associated with CTC phenotype in mCRC, analyzed with Ingenuity Pathways Analysis (IPA) software. (**C**) Gene ontology analysis (GOSlim) of CTC biological processes obtained by using Genecodis software.
(TIF)

Figure S3 **Principal gene interaction networks provided by IPA analysis.** (A) Cancer network. (B) Cellular movement and morphology network.
(TIF)

Table S1 **Patient clinico-pathological characteristics.** SD: Standard Deviation.
(DOC)

Table S2 **CTC specific genes found by microarray analysis.**
(XLS)

Table S3 **TaqMan qPCR probes characteristics.** Reference numbers refer to Applied Biosystems identification numbers. RefSeq refer to gene reference sequences. bp: base pairs.
(DOC)

Table S4 **Common genes between CTC profiling and liver metastasis specific genes (Lin et al).**
(DOC)

Methods S1 **Supporting methods.**
(DOC)
