# Supplementary figures and images for: Molecular Characterization of Circulating Tumor Cells in Human Metastatic Colorectal Cancer
Source: PLoS One. 2012 Jul 10;7(7):e40476. doi: 10.1371/journal.pone.0040476 (PMC3397799; doi:10.1371/journal.pone.0040476)

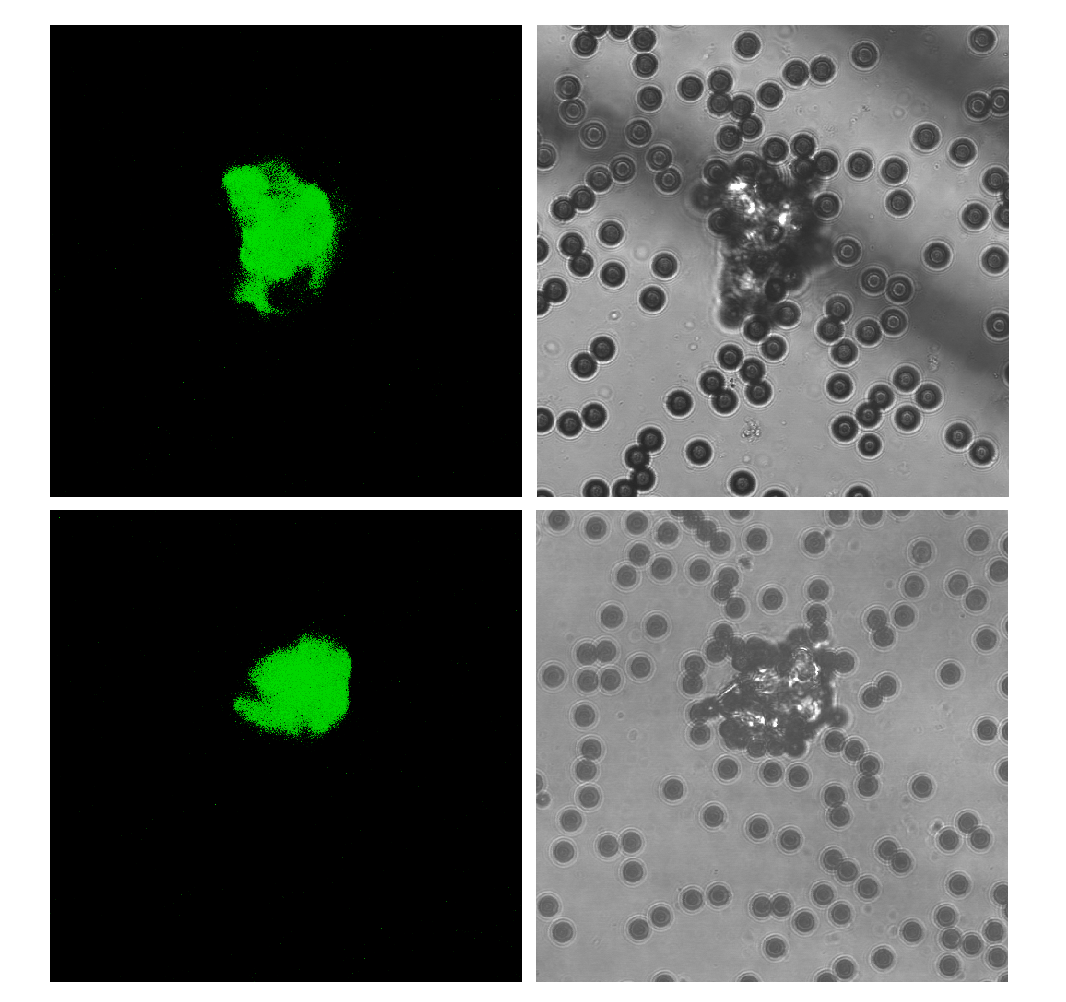

Supplement: Figure S1 — Representative images of epithelial cells isolated from mCRC patient’s blood. Cells were stained with a cocktail of antibodies against citokeratins (left panels) and visualized in clear field (right panels). (TIF) [file pone.0040476.s001.tif]

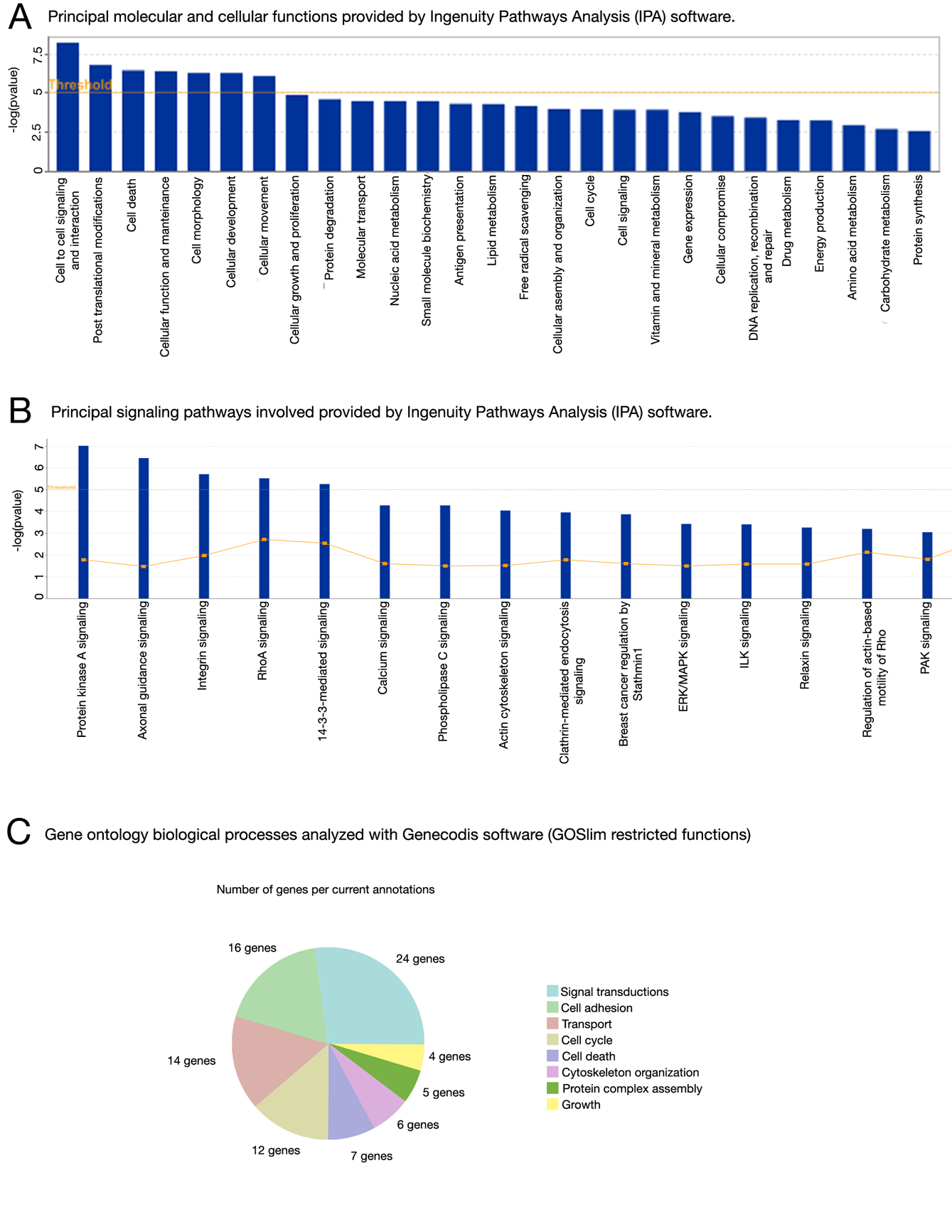

Supplement: Figure S2 — Gene interaction analysis. Principal molecular and cellular functions (A) and signaling pathways (B) associated with CTC phenotype in mCRC, analyzed with Ingenuity Pathways Analysis (IPA) software. (C) Gene ontology analysis (GOSlim) of CTC biological processes obtained by using Genecodis software. (TIF) [file pone.0040476.s002.tif]

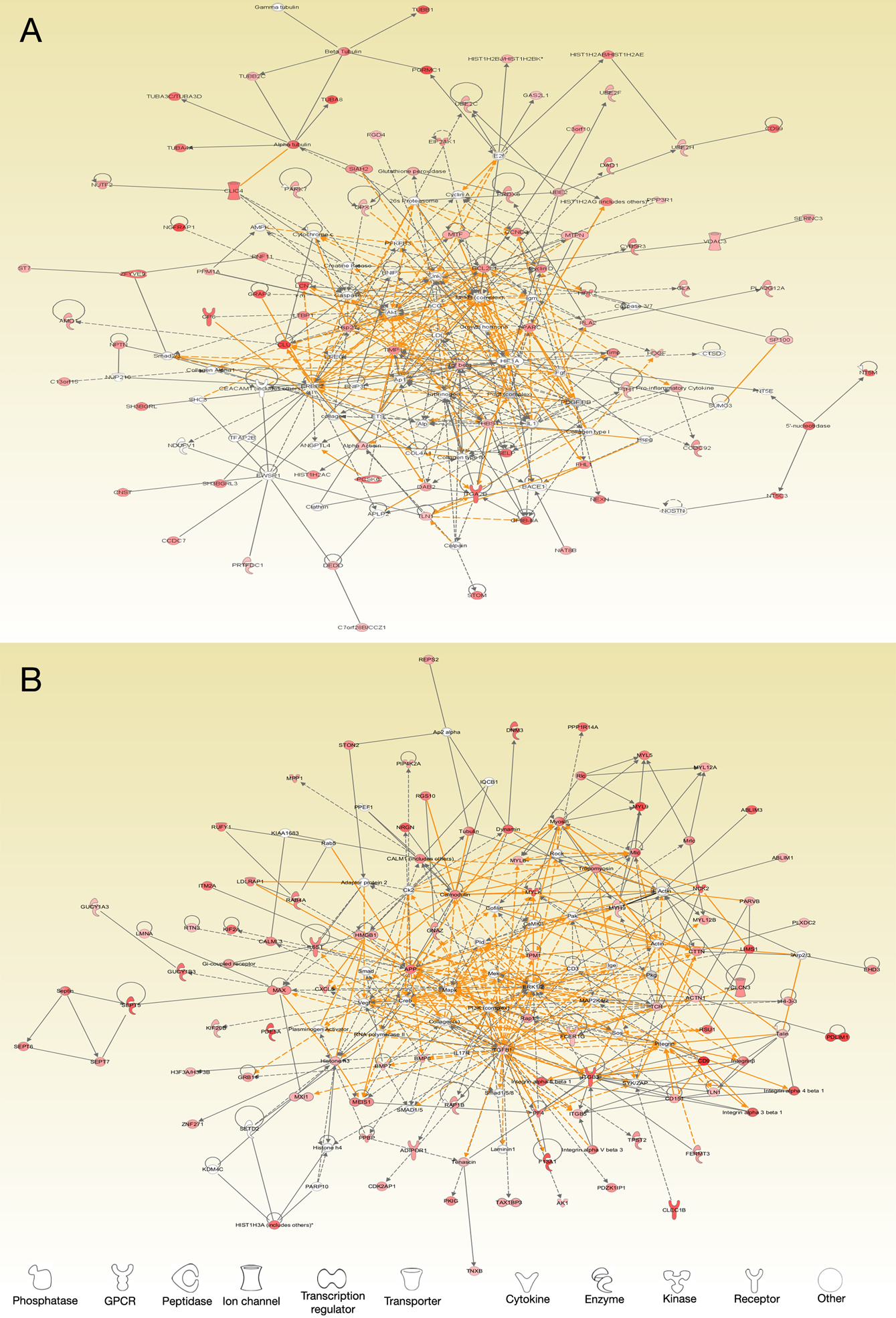

Supplement: Figure S3 — Principal gene interaction networks provided by IPA analysis. (A) Cancer network. (B) Cellular movement and morphology network. (TIF) [file pone.0040476.s003.tif]
