# Supplementary material for: Molecular Characterization of Circulating Tumor Cells in Human Metastatic Colorectal Cancer
Source: PLoS One. 2012 Jul 10;7(7):e40476. doi: 10.1371/journal.pone.0040476 (PMC3397799; doi:10.1371/journal.pone.0040476)
Supplement: Table S1 — Patient clinico-pathological characteristics. SD: Standard Deviation. (DOC) [file pone.0040476.s004.doc]

| **MICROARRAY PATIENTS** | | | | | | | | | | | | | |
| --- | --- | --- | --- | --- | --- | --- | --- | --- | --- | --- | --- | --- | --- |
| **Patient ID** | | | | | | | **Gender** | **Primary tumor** | | | **Metastasis** | | **Kras status** |
| Patient 1 | | | | | | | Male | Rectum adenocarcinoma | | | Hepatic | | Wild Type |
| Patient 2 | | | | | | | Male | Colon adenocarcinoma | | | Hepatic, omental | | Wild Type |
| Patient 3 | | | | | | | Female | Colon adenocarcinoma | | | Peritoneum | | Wild Type |
| Patient 4 | | | | | | | Male | Colon adenocarcinoma | | | Hepatic, adrenal | | Wild Type |
| Patient 5 | | | | | | | Male | Colon adenocarcinoma | | | Hepatic, peritoneum | | Undetermined |
| Patient 6 | | | | | | | Male | Colon adenocarcinoma | | | Hepatic | | Mutated |
| **qPCR VALIDATION PATIENTS** | | | | | | | | | | | |  | |
| **Age (years)** | | | | | | | | | | | |
|  | | | | | | Mean | | | 64.75 | | |
|  | | | | | | SD | | | 9.41 | | |
|  | | | | | | Range | | | 45-77 | | |
| **Gender** | | | | | | | | | **N** | **%** | |
|  | | | | | Male | | | | 13 | 65 | |
|  | | | | | Female | | | | 7 | 35 | |
| **Primary tumor location** | | | | | | | | | | | |
|  | | | | Colon | | | | | 17 | 85 | |
|  | | | | Rectum | | | | | 2 | 10 | |
|  | | | | Both | | | | | 1 | 5 | |
| **K-ras status** | | | | | | | | | | | |
|  | | | Wild Type | | | | | | 8 | 40 | |
|  | | | Mutated | | | | | | 6 | 30 | |
|  | | | Unknown | | | | | | 6 | 30 | |
| **pT** | | | | | | | | | | | |
|  | | | | pT_3_ | | | | | 14 | 70 | |
|  | | | | pT_4_ | | | | | 2 | 10 | |
|  | | | | pT_X_ | | | | | 4 | 20 | |
| **pN** | | | | | | | | | | | |
|  | | pN_0_ | | | | | | | 2 | 10 | |
|  | | pN_1_ | | | | | | | 6 | 30 | |
|  | | pN_2_ | | | | | | | 7 | 35 | |
|  | | pN_X_ | | | | | | | 5 | 25 | |
| **Number of metastatic sites** | | | | | | | | | | | |
|  | 1 | | | | | | | | 11 | 55 | |
|  | ≥2 | | | | | | | | 9 | 45 | |
| **Metastasis location** | | | | | | | | | | | |
|  | | Liver | | | | | | | 8 | 40 | |
|  | | Liver and other | | | | | | | 6 | 30 | |
|  | | Non liver | | | | | | | 6 | 30 | |
