# Supplementary material for: Molecular Characterization of Circulating Tumor Cells in Human Metastatic Colorectal Cancer
Source: PLoS One. 2012 Jul 10;7(7):e40476. doi: 10.1371/journal.pone.0040476 (PMC3397799; doi:10.1371/journal.pone.0040476)
Supplement: Table S3 — TaqMan qPCR probes characteristics. Reference numbers refer to Applied Biosystems identification numbers. RefSeq refer to gene reference sequences. bp: base pairs. (DOC) [file pone.0040476.s006.doc]

| Gene tested | Reference | RefSeq | Amplicon length (bp) |
| --- | --- | --- | --- |
| PTPRC | Hs00894734_m1 | NM_002838.3 | 70 |
| GAPDH | Hs99999905_m1 | NM_002046.3 | 122 |
| RSU1 | Hs00757864_m1 | NM_152724.2 NM_012425.3 | 80 |
| LIMS1 | Hs99757864_m1 | NM_001193482.1 NM_001193483.1 NM_001193484.1 NM_001193485.1 NM_001193488.1 | 75 |
| BMP6 | Hs01099594_m1 | NM_001718.4 | 108 |
| TGFB1 | Hs00998133_m1 | NM_000660.4 | 57 |
| TIMP1 | Hs00171558_m1 | NM_003254.2 | 104 |
| CD9 | Hs00233521_m1 | NM_001769.3 | 72 |
| TLN1 | Hs00196775_m1 | NM_006289.3 | 113 |
| ITGB5 | Hs00174435_m1 | NM_002213.3 | 78 |
| VCL | Hs00243320_m1 | NM_014000.2 NM_003373.3 | 73 |
| CLU | Hs00156548_m1 | NM_203339.1 NM_001171138.1 NM_001831.2 | 65 |
| APP | Hs01552283_m1 | NM_201413.2 NM_201414.2 NM_001136129.2 NM_001136130.2 NM_000484.3 | 152 |
