# Supplementary material for: Molecular Characterization of Circulating Tumor Cells in Human Metastatic Colorectal Cancer
Source: PLoS One. 2012 Jul 10;7(7):e40476. doi: 10.1371/journal.pone.0040476 (PMC3397799; doi:10.1371/journal.pone.0040476)
Supplement: Table S4 — Common genes between CTC profiling and liver metastasis specific genes (Lin et al). (DOC) [file pone.0040476.s007.doc]

| **Gene name** | **Description** |
| --- | --- |
| BMP6 | Homo sapiens bone morphogenetic protein 6 |
| BMP7 | Homo sapiens bone morphogenetic protein 6 |
| BRD3 | Homo sapiens bromodomain containing 3 |
| CMAH | Homo sapiens cytidine monophosphate-N-acetylneuraminic acid hydroxylase (CMP-N-acetylneuraminate monooxygenase) pseudogene. |
| F13A1 | Homo sapiens coagulation factor XIII, A1 polypeptide |
| F2R | Homo sapiens coagulation factor II (thrombin) receptor |
| FCER1G | Homo sapiens Fc fragment of IgE, high affinity I, receptor for; gamma polypeptide |
| GNAS | Homo sapiens GNAS complex locus (GNAS), transcript variant 7 |
| GUCY1A3 | Homo sapiens guanylate cyclase 1, soluble, alpha 3 |
| HIST2H2BE | Homo sapiens histone cluster 2, H2be |
| HMGB1 | Homo sapiens high-mobility group box 1 |
| ITGB3 | Homo sapiens integrin, beta 3 (platelet glycoprotein IIIa, antigen CD61) |
| ITM2A | Homo sapiens integral membrane protein 2A |
| MITF | Homo sapiens microphthalmia-associated transcription factor |
| MPP1 | Homo sapiens membrane protein, palmitoylated 1 |
| NAPG | Homo sapiens N-ethylmaleimide-sensitive factor attachment protein, gamma |
| PDE5A | Homo sapiens phosphodiesterase 5A, cGMP-specific |
| RAB27B | Homo sapiens RAB27B, member RAS oncogene family |
| REPS2 | Homo sapiens RALBP1 associated Eps domain containing 2 |
| SLC39A3 | Homo sapiens solute carrier family 39 (zinc transporter), member 3 |
| SLC40A1 | Homo sapiens solute carrier family 40 (iron-regulated transporter), member 1 |
| SNRPN | Homo sapiens small nuclear ribonucleoprotein polypeptide N |
| SPARC | Homo sapiens secreted protein, acidic, cysteine-rich (osteonectin) |
| STOM | Homo sapiens stomatin |
| THBS1 | Homo sapiens thrombospondin 1 |
| TIMP1 | Homo sapiens TIMP metallopeptidase inhibitor 1 |
| TPM1 | Homo sapiens tropomyosin 1 |
| TUBA4A | Homo sapiens tubulin, alpha 4a |
| XK | Homo sapiens X-linked Kx blood group (McLeod syndrome) |
| YWHAZ | Homo sapiens tyrosine 3-monooxygenase/tryptophan 5-monooxygenase activation protein, zeta polypeptide |
