# Supplementary material for: Molecular Characterization of Circulating Tumor Cells in Human Metastatic Colorectal Cancer
Source: PLoS One. 2012 Jul 10;7(7):e40476. doi: 10.1371/journal.pone.0040476 (PMC3397799; doi:10.1371/journal.pone.0040476)
Supplement: Methods S1 — Supporting methods. (DOC) [file pone.0040476.s008.doc]

**Supporting methods**

**Immunofluorescence.**

Immunoisolated cells were resuspended in PBS containing 1%BSA, 2mM EDTA. Cell suspensions were applied to the surface of a glass slide and allowed to dry. Cells were then labeled with a cocktail of antibodies against citokeratins 8, 18 and 19 conjugated to phycoerythrin. Sequential images were taken by confocal microscopy.

**Recovery experiments.**

Increasing numbers of HT29 cells (10, 100, 1000 and 10000) were resuspended in RNAlater solution (Ambion), with and without a previous step of immunoisolation. RNA was extracted with the Qiamp Viral Kit (Qiagen) and cDNA was synthetized with SuperScriptIII (Invitrogen). Complementary DNA was pre-amplified (TaqMan Preamp, Applied Biosystems) previous to the GAPDH RTqPCR (TaqMan chemistry, Applied Biosystems). Recovery rate was calculated as the median percentage of immunoisolated cells compared to the corresponding control without isolation. The experiments were performed at least in duplicate.

**Gene Expression Analysis**

**RNA extraction from immunoisolated CTCs, whole transcriptome amplification and array hybridization.** Total RNA from CTCs was extracted with the QIAmp viral RNA mini kit (Qiagen), specifically designed for very low cellularity samples. Cell-bead complexes were loaded directly onto the isolation columns. No RNA carrier was used in order to avoid interferences in further amplification steps. Once extracted, RNA samples were DNAseI treated (RNAse free DNase set, Qiagen) for 10 minutes at room temperature and purified with the QIAquick® PCR purification kit (Qiagen). Purified RNA was next subjected to a whole transcriptome amplification reaction (WTA2, Sigma Aldrich), following manufacturer instructions. The maximum RNA amount permitted was amplified (20 reaction cycles), Cy3 labeled and hybridized onto Agilent 4x44k gene-expression arrays. Upon hybridization, signal was captured and processed using an Agilent scanner (G2565B, Agilent Technologies). The scanner images were segmented by the Agilent Feature Extraction Software (v9.5) with the protocol GE1-v5_95. Extended dynamic range implemented in the Agilent software was applied to avoid saturation in the highest intensity range. The Agilent feature extraction was used as raw data for further pre-processing. The processed signal (gProcessed-Signal) value was chosen for the statistical analysis instead of the signal with subtracted background (gBGSubSignal) since it produces lower average coefficient of variation (CV) in Spike-In and gene replicates (Klebanov & Yakovlev, 2007 *Biol Direct* 2:9; Zahurak et al., 2007 *BMC Bioinformatics* 8:142). Spatial Detrend correction was applied using the Agilent Feature Extraction algorithm. The following features and/or genes which did not conform to the established quality criteria were filtered: (a) non-uniform pixel distributed outliers and population replicate outliers according to the default Agilent feature extraction criteria; (b) spots not differentiated from background signal (as estimated for each spot); (c) spots in the range of negative controls. Gene expression data is accessible at the NCBI Gene Expression Omnibus (GEO) database (Accession Number: GSE31023).

**Gene expression data analysis.** Genes characterising the CTC population isolated from mCRC patients were identified by using the MeV v4.7 (Multiexperiment Viewer) software (TM4 Microarray Software Suite; Saeed et al., 2003 *Biotechniques* 34:374-378; Saeed et al., 2006 *Methods Enzymol* 411:134-193). One class Significance Analysis for Microarrays (SAM) algorithm was applied for the selection of candidate genes with stringent criteria to obtain significance within a slide and among all slides. First, data transformed in ratios and base 2 logarithms between mCRC samples and the unspecific healthy controls within a slide must fit Delta=1 and Mean False Discovery Rate (FDR) <0,5% values; and second, ratios from every mCRC sample normalized to mean unspecific healthy controls must fit Delta=2,3 and Mean FDR<0,75%. These analyses rendered a list of 386 genes. In addition, we added a set of genes exclusively expressed in mCRC patients, presenting positive signal in at least five patients but detectable and low signal in a maximum of one healthy control. All these criteria resulted in a final list of 410 genes specifically characterising the CTC population from mCRC patients.

Hierarchical clustering (HCL) was performed with these significantly expressed genes using MeV. Pearson correlation was chosen as distance metric, using absolute distance and complete linkage clustering parameters in MeV options.

Gene sets were firstly analyzed with Ingenuity Pathway Software (IPA) for gene networks generation and to provide a global view of the signalling pathways characterizing CTCs from mCRC patients. Out of the 410 significant genes, 368 were annotated genes and thus identified by IPA. Fisher’s exact test was applied to calculate p values of biological related functions. A p value of 10^-5^ was established as threshold for the selection of molecular and cellular functions, diseases/disorders and physiological system and development related functions. Gene interaction networks were generated by merging the four most relevant networks that included functions and biological processes of interest. In parallel, Gene Ontology analyses were done using the GENECODIS 2.0 open access software (Carmona-Saez et al., 2007 *Genome Biol* 8:R3; Nogales-Cadenas et al., 2009 *Nucleic Acids Res* 37:W317-322). GOSlim cut-down version of the GO ontologies was used as it contains only a reduced subset of terms in the whole GO, and gives a broad overview of the ontology content. Biological processes implicated were analyzed by this methodology, using hypergeometrical statistical tests and FDR p-value corrections, as indicated in the software.
